# Regulation of HIF-1 by MicroRNAs in Various Cardiovascular Diseases

**DOI:** 10.2174/1573403X19666230330105259

**Published:** 2023-07-17

**Authors:** Vahideh Tarhriz, Leila Abkhooie, Mostafa Moradi Sarabi

**Affiliations:** 1 Molecular Medicine Research Center, Biomedicine Institute, Tabriz University of Medical Sciences, Tabriz, Iran;; 2 Faculty of Pharmacy, Tabriz University of Medical Sciences, Tabriz, Iran;; 3 Razi Herbal Medicines Research Center, Lorestan University of Medical Sciences, Khorramabad, Iran;; 4 Department of Medical Biotechnology, Faculty of Medicine, Lorestan University of Medical Sciences, Khorramabad, Iran;; 5 Department of Biochemistry and Genetics, School of Medicine, Lorestan University of Medical Sciences, Khorramabad, Iran

**Keywords:** Hypoxia-Inducible factor 1 (HIF-1), miRNAs, cardiovascular diseases, atherosclerosis, myocardial infarction, cardiac hypertrophy

## Abstract

Today, we see an increase in death due to cardiovascular diseases all over the world, which has a lot to do with the regulation of oxygen homeostasis. Also, hypoxia-inducing factor 1 (HIF-1) is considered a vital factor in hypoxia and its physiological and pathological changes. HIF-1 is involved in cellular activities, including proliferation, differentiation, and cell death in endothelial cells (ECs) and cardiomyocytes. Similar to HIF-1α, which acts as a protective element against various diseases in the cardiovascular system, the protective role of microRNAs (miRNAs) has also been proved using animal models. The number of miRNAs identified in the regulation of gene expression responsive to hypoxia and the importance of investigating the involvement of the non-coding genome in cardiovascular diseases is increasing, which shows the issue's importance. In this study, the molecular regulation of HIF-1 by miRNAs is considered to improve therapeutic approaches in clinical diagnoses of cardiovascular diseases.

## 1 INTRODUCTION

The cardiovascular system delivers oxygen to the respiratory tissues, and to date, a wide variety of adaptive cardiovascular reactions to hypoxia have been identified. Hypoxia-inducible factors (HIFs) are the key oxygen-sensing mechanism defined by the post-translational hydroxylation of HIFα *via* a set of 2-oxoglutarate-dependent dioxygenases [1-4].

The HIF factor is a complex of α and β heterodimers, and in humans, there are 3 isoforms of the HIFα regulatory dimerization partner, each of which is a target for oxygen-sensitive dioxygenases [1, 5, 6]. The HIF-1α protein is unstable to oxygen and is controlled *via* oxygen at the post-translational level by hydroxylation of prolyl hydroxylase domain (PHD) proteins at specific proline residues. While the β subunit is constitutively expressed, it does not play a role in oxygen detection [7-9].

**Fig (1):**
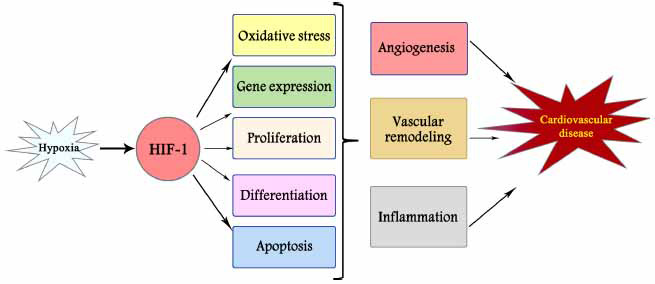
HIF-1 regulates hypoxia-induced physiological and pathological changes including oxidative stress, angiogenesis, vascular remodeling, and inflammatory responses in the cardiovascular system.

HIF-1 is involved in cellular activities such as oxidative stress, angiogenesis, and immune activities (Fig. **1**) [10-13]. MiRNAs are the most studied subtype of ncRNAs. MiRNAs are small molecules of 20 to 26 nucleotides with a highly conserved single-stranded RNA sequence. MiRNAs regulate many biological processes and these regulatory effects are largely achieved by destabilizing target mRNAs or inhibiting translation [14]. Continuing studies show that HIF-1α and mRNAs play important roles in cardiovascular disease (CVD). In this review, we studied the reciprocal regulation between HIF-1α and mRNAs, as well as their effects on various CVD. Also, we expressed the therapeutic potential of targeting hypoxia-inducible factor signals in heart diseases.

## 2 EFFECT OF THE HIF BY MIRS IN CARDIOVASCULAR DISEASE

Regulation of oxygen homeostasis is key in cardiovascular disease, and changes due to hypoxia can be regulated by factors such as HIF-1 [8, 15]. Adjusting hypoxia effectively treats a cardiovascular disease associated with hypoxia [8, 10, 16]. MiRNAs are involved in regulating the expression of the HIF-1 factor, and studies have shown that reducing miRNAs such as miR-199a increases HIF-1 expression during hypoxia [16, 17]. Also, the expression level of HIF-1α and Sirtuin 1 (Sirt1) increases with the removal of miR-199a during normoxia, resulting in complications of hypoxia [16, 17]. We are interested in understanding the molecular mechanism of HIF-1 and how it regulates this factor in regulating the process of hypoxia and related heart diseases (Table **1**) [17, 18].

### 2.1 HIF and miRs in Atherosclerosis

Atherosclerosis (AS) is one of the major causes of mortality in heart diseases, and atherosclerotic plaques are formed due to immune system response and inflammatory reactions [27, 28]. Also, miRNAs have an essential role during inflammatory processes and their relationship with genes such as HIF-1α can be considered to study the process of atherosclerosis [20, 29, 30].

HIF-1α rises the expression of hypoxia-induced angiogenesis genes by miRNA [19, 20]. For example, Bartoszewska *et al.* showed that HIF-1 expression is reduced by miR-429 in the early stages of hypoxia before HIF-2 activation. Also, the conversion of HIF-1 to HIF-2 occurs in endothelial cells during chronic hypoxia [31]. Studies show that in vascular endothelial cells, the expression of hypoxia-responsive microRNAs (HRMs), including Let-7 and miR-103/107 is increased by HIF1α [19, 32, 33]. Subsequently, Chen and colleagues demonstrated that HIF1α and its target argonaute 1 (AGO1) induce these HRMs [19].

**Fig. (2):**
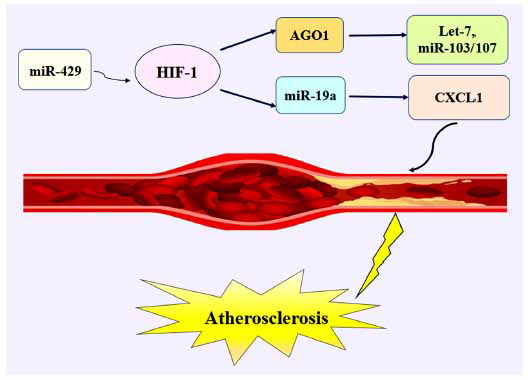
Regulation mechanism of HIF-1 in Atherosclerosis.

HIF-1α can up-regulate the expression of CXCL1 by miR-19a. The accumulation of (C - X - C motif) ligand 1 (CXCL1) leads to the attachment of monocytes to dysfunctional endothelial cells (ECs) and results in the gathering of macrophages, which is associated with increased arterial inflammation in atherosclerotic disease (Fig. **2**) [20].

### 2.2 HIF and miRs in Pulmonary Hypertension

In pulmonary hypertension (PH), we see an extreme increase in vascular cells and an increase in the resistance of pulmonary vessels due to the gathering of inflammatory cells in the vessel wall, which can result in heart failure and death. Studies show that regulating HIF factor isoforms and their conditional and specific knockout can be a pivotal character in the development of PH and in identifying the disease process [34].

HIF not only performs a role in the development of PH disease, but it is also effective in hypoxia caused by other lung diseases such as COPD or exposure to high altitude, *etc*. [34]. Recent findings indicate that the cell type and tissue-specific regulation of HIF isoforms are regulated in pulmonary artery smooth muscle cells (PASMCs) because enhanced expression of HIF-2α has been detected in the lung tissue of patients [34, 35]. Also, the expression of HIF-1α increases in diseases such as chronic thromboembolic PH by affecting the genes vascular endothelial growth factor (VEGF) and erythropoietin (EPO) [21].

Zeng *et al.* showed that in hypoxia, HIF-1α increases the expression of miR-322. Also, miR-322 has positive feedback on HIF-1α, and the effect of miR-322 on BMPR1a and smad5 stimulates the proliferation and migration of smooth muscle cells, and pulmonary vascular remodeling related to PH occurs [22]. Gorospe *et al.* displayed that although a HIF response element (HRE) is foreseen in the miR-21 promoter, the level of this miRNA is amplified by HIF in pulmonary artery endothelial cells [36]. Deletion of miR-17~92 in smooth muscle cells reduces the expression of HIF-1α and decreases cell proliferation through PASMC proliferation *via* prolyl hydroxylases (PHDs), followed by a reduction in pulmonary blood pressure caused *via* hypoxia [23]. In conclusion, the HIF factor can be considered a tool for the treatment of patients with PH.

### 2.3 HIF and miRs in Cardiac Hypertrophy

Cardiac hypertrophy involves increased heart size, increased protein synthesis, and higher sarcomere organization, which can lead to cardiac dysfunction. Also, fetal gene reactivation, abnormality of energy metabolism, and increased protein synthesis can be associated with cardiac hypertrophy [37-39].

Recently, a subset of muscle-specific miRNAs, such as miR-1 and miR-133, have been displayed to perform a central part in muscle cell proliferation and differentiation [40-42]. Examining the miRNA profile of chronic heart failure (CHF) patients shows an amplified level of miR-217 expression. Also, the progression of cardiac hypertrophy is associated with increased expression of miR-217, which is due to the expression of tension homolog deleted on chromosome ten (PTEN) [37, 43].

The expression level of miR-29c is controlled by HIF-1, and miR-29c can downregulate SERCA2 expression in a transgenic overexpression scheme [14]. MiR-92 and miR-100 are involved in cardiac hypertrophy and studies show that the level of miR-92 expression is decreased, while the increase of miR-100 can negatively affect the expression of mature α-MHC and sarco (endo) plasmic reticulum ATPases (SERCA) genes. Likewise, miR-100 increases the expression of fetal ANF and β-MHC genes and has a positive effect on their expression [40]. Rane *et al.* showed that miR-199a expression was downregulated following insulin receptor-induced Akt pathway activation in neonatal rat cardiomyocytes after isoproterenol stimulation. Subsequently, miR-199a increases the expression of HIF-1α and sirtuin (Sirt1), a histone deacetylase [24, 44, 45].

The reduction of miR-199a can inhibit the hypoxia-induced proapoptotic pathways mediated by HIF-1α under hypoxia conditions, and also the upregulation of miR-199a with an adenoviral vector increases cell hypertrophy *in vitro* [24]. Similar to miR-199b, miR-23a can be prepared by the calcineurin/NFAT path under the influence of isoproterenol-induced hypertrophy [24]. The expression of miR-21 and miR-18b was inhibited *via* locked nucleic acid (LNA)-modified antisense oligonucleotides in cardiac cells by Tatsuguchi *et al.* After that, miR-21 expression increased and miR-18b decreased in cardiomyocytes. These results show that miR-18b and miR-21 can be effective in cardiomyocyte hypertrophy [25].

### 2.4 HIF and miRs in Diabetic Cardiomyopathy

Almost 80% of the deaths of diabetic patients occur due to Diabetic heart disease (DHD) and although it starts in the early stages of diabetes, it is not detectable and usually has no symptoms until the last stages of the disease. Therefore, it is important to find early diagnostic methods for this purpose, and miRNAs (miRs) can be studied as biomarkers in the regulation of gene expression and other cellular processes [46-48].

The studies showed the transcription of miRs including miR-34b, miR-34c, miR-199b, miR-210, miR-223, and miR-650 in sampling from the left ventricle of heart attack patients with diabetes (D-HF) and without Diabetes (ND-HF) is different and HIF expression is increased in D-HF patients [26].

Heather *et al.* showed that in hypoxia and high blood glucose, the expression level of miR-199a / b increases in cultured myocardium and endothelial cells. This increase in the expression of miR-199b is the result of the initiation of calcineurin/nuclear factor of activated T cells path [49].

In normal people, the HIF signaling pathway is activated in conditions of oxygen deficiency and provides oxygen-independent ATP synthesis. In diabetic subjects, the HIF expression level is increased and the HIF-dependent hypoxia response is decreased. Also, the HIF/HIF pathway by reactive oxygen species can lead to an increase in oxidative stress. Also, the oxidative stress by miRNA and their regulated genes can paradoxically decrease HIF expression after stroke [46]. Under hypoxic conditions, miR-210 is affected by HIF-1α and downregulates PARK7, a redox-responsive cellular protective protein, in diabetic HF patients [50]. In D-HF patients, it has been observed that miR-210 protects heart cells by inhibiting apoptosis [26]. Decreased expression of miR-1 / 133a has been reported in cardiac hypertrophy and fibrosis, while increased miR-1 under hyperglycemic conditions can induce apoptosis in cardiac cells. Also, studies show that the expression level of miR-1 and miR-133 can be used to evaluate the complications of diabetes in the serum of patients with type 2 diabetes with myocardial steatosis [51-53].

## 3 PERSPECTIVES ON HIF-1Α AND NCRNAS IN CLINICAL PRACTICE

Ischemia can be one of the causes of myocardial infarction, and blood reperfusion to the ischemic area is considered a treatment method. However, this treatment method can cause ischemic reperfusion injury (IRI) and cell death due to the quick growth of ROS. To prevent these damages, cells can be exposed to short cycles of ischemia-reperfusion before the longer and chronic phase of IRI, and it is theorized that the HIF signaling path may contribute to the fundamental apparatuses of ischemic preconditioning. Studies of the HIF pathway provide a cellular solution to hypoxic stress and can be considered for the treatment of ischemic patients [54-56].

The expression of HIF-1 can form collateral vessels and lead to severe consequences in atherosclerosis patients, and for this reason, it is not a suitable option for the treatment of these patients [57]. Until now, most of the studies have been done on HIF-α subunits, and perhaps by increasing the awareness of molecular pathways and the role of other subunits such as HIF-β, effective therapeutic methods can be achieved in the treatment of heart diseases for example atherosclerosis [54, 57, 58].

Other research shows that PH disease, which is the result of the proliferation of pulmonary artery smooth muscle cells and endothelial cells by HIF-1 and HIF-2, and inhibiting these factors can be considered as a therapeutic strategy [59, 60]. In general, more research should be done to find more details about the relationship of HIF and its subunits with cellular processes and its role in the development of heart diseases to provide us with more effective prospects in the treatment of these diseases.

## CONCLUSION

In this review, we studied the collected data on HIF and the complex molecular network under its control in hypoxic conditions. Understanding the mentioned molecular pathways can be used in applying more efficient treatment methods for cardiovascular diseases.

## Figures and Tables

**Table 1:** HIF in cardiovascular disease.

**Diseases**	**microRNAs**	**Effects**	**References**
**Atherosclerosis**	Let-7 and miR-103/107	HIF1α and its target argonaute 1 (AGO1) induce these HRMs (Let-7 and miR-103/107).	[19]
	miR-19a	HIF-1α can up-regulate the expression of CXCL1 by miR-19a and results in the gathering of macrophages.	[20]
**Pulmonary hypertension (PH)**		HIF-1α increases by affecting the genes vascular endothelial growth factor (VEGF) and erythropoietin (EPO) in PH.	[21]
	miR-322	HIF-1α increases the expression of miR-322 and stimulates the BMPR1a and smad5. As a result, the proliferation and migration of smooth muscle cells occur in PH.	[22]
	miR-17~92	Deletion of miR-17~92 in smooth muscle cells reduces the expression of HIF-1α and decreases cell proliferation through PASMC proliferation *via* prolyl hydroxylases (PHDs).	[23]
**Cardiac hypertrophy**	miR-29c	HIF-1 controls the miR-29c which can downregulate SERCA2 expression in a transgenic overexpression scheme.	[14]
	miR-199a	miR-199a increases the expression of HIF-1α and sirtuin (Sirt1), a histone deacetylas.	[24]
	miR-21 / miR-18b	Upregulation of miR-21 and downregulation of miR-18b can be effective in cardiomyocyte hypertrophy.	[25]
**Diabetic cardiomyopathy**	miR-210	HIF-1α downregulates PARK7 by miR-210 and also, miR-210 protects heart cells by inhibiting apoptosis.	[26]

## LIST OF ABBREVIATIONS

EPO: Erythropoietin
VEGF: Vascular Endothelial Growth Factor
Pasmcs: Pulmonary Artery Smooth Muscle Cells
PH: Pulmonary Hypertension
